# Positioning of the SCRAMBLED receptor requires UDP-Glc:sterol glucosyltransferase 80B1 in *Arabidopsis* roots

**DOI:** 10.1038/s41598-017-05925-6

**Published:** 2017-07-18

**Authors:** Victoria G. Pook, Meera Nair, KookHui Ryu, James C. Arpin, John Schiefelbein, Kathrin Schrick, Seth DeBolt

**Affiliations:** 1 Department of Horticulture, University of Kentucky, Lexington, KY 40546 USA; 2 Department of Molecular, Cellular, and Developmental Biology, University of Michigan, Ann Arbor, MI 48109 USA; 3 Division of Biology, Kansas State University, Manhattan, KS 66506 USA

## Abstract

The biological function of sterol glucosides (SGs), the most abundant sterol derivatives in higher plants, remains uncertain. In an effort to improve our understanding of these membrane lipids we examined phenotypes exhibited by the roots of Arabidopsis *(Arabidopsis thaliana*) lines carrying insertions in the UDP-Glc:sterol glucosyltransferase genes, *UGT80A2* and *UGT80B1*. We show that although *ugt80A2* mutants exhibit significantly lower levels of total SGs they are morphologically indistinguishable from wild-type plants. In contrast, the roots of *ugt80B1* mutants are only deficient in stigmasteryl glucosides but exhibit a significant reduction in root hairs. Sub-cellular investigations reveal that the plasma membrane cell fate regulator, SCRAMBLED (SCM), is mislocalized in *ugt80B1* mutants, underscoring the aberrant root epidermal cell patterning. Live imaging of roots indicates that *SCM:GFP* is localized to the cytoplasm in a non cell type dependent manner instead of the hair (H) cell plasma membrane in these mutants. In addition, we provide evidence for the localization of the *UGT80B1* enzyme in the plasma membrane. These data lend further support to the notion that deficiencies in specific SGs are sufficient to disrupt normal cell function and point to a possible role for SGs in cargo transport and/or protein targeting to the plasma membrane.

## Introduction

Phytosterols, exceptional in their diversity, serve as structural components of biomembranes, regulating their fluidity and permeability^1^. Dominated by sitosterol, campesterol and stigmasterol, the plant sterol profile also comprises low levels of brassicasterol and cholesterol^2^. In Arabidopsis, mutations in genes for sterol biosynthesis enzymes such as *FACKEL* (*FK*) (sterol C-14 reductase), *STEROL METHYLTRANSFERASE 1* (*SMT1*) (C-24 sterol methyltransferase), *HYDRA1* (*HYD1*) (sterol C8-7 isomerase) and *COTYLEDON VASCULAR PATTERN1* (*CVP1*)(C-28 sterol methyltransferase) result in severe developmental phenotypes^3–7^. In addition, loss of epidermal cell file organization in the root was observed in mutants for *FK/HYDRA2* (*HYD2*)^5, 6^, *SMT1* and the double mutant for *SMT2* and *SMT3* ^3^. In *smt2 smt3* mutants, visible defects in oriented cell plate formation and polarized growth in the root are associated with defects in endocytic recycling^8^. Exogenous application of the ethylsterols sitosterol and stigmasterol, but not the methylsterol campesterol, partially rescued lateral root growth defects^8^, implicating specific sterols in this process.

Among various sterol derivatives, the most abundant are steryl glucosides (SGs)^9–12^. First discovered as ‘ipuranol’ from the bark of the olive tree and *Ipomoea purpurea* ^13, 14^, SGs result from the decoration of the C3-hydroxyl group of a sterol with a glucose sugar moiety, a reaction catalyzed by glucosyltransferases. The genome of Arabidopsis contains two genes coding for UDP-Glc:sterol-glucosyltransferases, *UGT80A2* and *UGT80B1*, and studies of mutant lines indicate that they are only partially redundant^15, 16^. While *UGT80A2* appears to be responsible for the generation of the bulk of the SGs in aerial tissue and seeds, it is *ugt80B1* mutants (and double mutants) that exhibit striking morphological phenotypes^15, 16^.

In their 2009 study, DeBolt *et al*.^15^ showed that *ugt80B1* single mutants and *ugt80A2,ugt80B1* double mutants exhibit growth defects during embryogenesis. Additional findings included the depletion of cutin and suberin from the seed coat, the display of a transparent testa phenotype and altered cell morphology. In contrast, *ugt80A2* single mutants displayed no obvious defects in plant growth. Lipid monomer analysis by gas chromatography coupled with mass spectrometry on seed coat cells of double mutants revealed a reduction in lipid polyesters and led to speculation as to whether SGs play a role in the trafficking of cargo molecules.

The apparent mismatch in the phenotypes of *ugt80A2* and *ugt80B1* mutants led Stucky *et al*.^16^ to investigate the substrate preferences of the two enzymes. *In vitro* assays suggest that UGT80B1 prefers brassicasterol as a substrate. These data combined with the sterol profile of *ugt80B1* mutants provide evidence for a preference for sterol substrates with unbranched side chains, leading Stucky *et al*.^16^ to propose a more specialized role for UGT80B1. UGT80A2, on the other hand, was able to utilize sitosterol, campesterol and stigmasterol *in vitro* and appears to be the dominant enzyme for SG production. Stucky *et al*.^16^ propose that the phenotypes associated with *ugt80B1* mutants result from deficiencies in specific minor compounds, rather than a reduction in total SGs. This proposition is supported by work by Grosjean *et al*.^17^ in which an environment-sensitive probe was used to investigate the level of membrane order in vesicles of varied lipid compositions. The study showed that membrane organization is influenced by the composition of different free and conjugated sterols and that individual types of sterols participate in membrane ordering in additive or subtractive ways.

In the present study we examine epidermal pattern formation in the root as an easily accessible aspect of Arabidopsis development that is dependent on signaling across membranes. In the current model (reviewed in Grierson *et al*.^18^), a positional signal is received by SCRAMBLED (SCM), which is a membrane-associated leucine-rich repeat receptor-like kinase (LRR-RLK) that initiates the differential expression of cell fate regulators^19^. Cells that contact the anticlinal cell walls of two underlying cortical cells develop root hairs (H cells, trichoblasts) and those remaining form non-hair cells (N cells, atrichoblasts)^20^. Downstream of SCM, WEREWOLF (WER) forms a transcription factor complex with GL3/EGL3/MYC1 and TTG that leads to the expression of the homeodomain leucine-zipper transcription factor GLABRA2 (GL2)^21–24^. GL2 inhibits ROOT HAIR DEFECTIVE 6 (RHD6), a positive regulator of H cell differentiation genes, while promoting N cell differentiating genes. In H cells, the expression of WER is inhibited, allowing CPC/TRY/ETC to form a transcriptional regulatory complex with GL3/EGL3/MYC1 and TTG. This leaves RHD6 uninhibited, positively regulating H cell differentiation genes.

Given that inter- and intracellular crosstalk is required for root epidermal cell patterning, we hypothesized that Arabidopsis lines carrying mutations in UDP-Glc:sterol glucosyltransferase genes may exhibit patterns of H and N cells that deviate from wild-type plants. We therefore investigated this phenotype in UDP-Glc:sterol glucosyltransferase mutants at both the whole organism and sub-cellular levels. Morphological assessment of *ugt80B1* mutants show that they exhibit aberrant root hair patterning and sub-cellular confocal microscopy indicates that this could be explained by the mislocalization of cell fate regulators, lending support to the hypothesis that SGs may influence intracellular communication.

## Results

### Sterol glucoside profiling

Mutations in genes coding for UDP-Glc:sterol glucosyltransferases have previously been shown to reduce the amount of SG present in seed, silique and leaf tissues^15, 16, 25^. In this study the sterol glucoside profiles of roots from *ugt80B1* mutants, *ugt80A2* mutants and *ugt80A2,B1* double mutants were measured. SGs were also examined in root tissue from wild type and *fk-J3158* mutants as controls. The *fk-J3158* mutant allele represents a weak allele of the *FK* (sterol C-14 reductase) gene^26^, and it was chosen due to the severity of the phenotype and difficulty in obtaining root material from *fk* null mutants.

**Figure 1:**
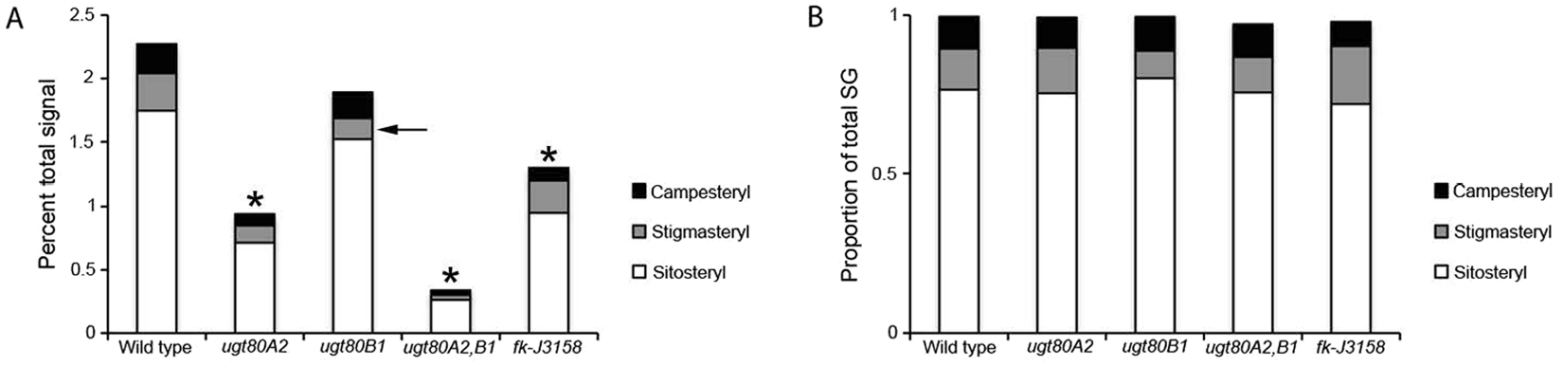
Steryl glucoside (SG) content in roots of wild-type and mutant lines. (**A**) Total steryl glucosides (SG), and sitosteryl, stigmasteryl, and campesteryl glucosides were quantified as percent total signal by ESI-MS/MS for wild type, *ugt80A2*, *ugt80B1*, *ugt80A2,B1* and *fk-J3158*. Significant reductions of SGs in comparison to wild-type levels were found in *ugt80A2*, *ugt80A2,B1* and *fk-J3158* (*p* < 0.001, T-test; indicated by asterisks). The *ugt80B1* mutant showed a significant reduction in stigmasteryl glucosides only (*p* < 0.001, T-test; indicated by arrow). (**B**) Campesteryl, stigmasteryl and sitosteryl glucosides as a proportion of total SG in wild-type and mutant lines. Stigmasteryl glucosides make up disproportionately less of the total signal in *ugt80B1* mutants when compared to wild type (*p* < 0.01, Dunnett’s Test). Note that the sum of these proportions is slightly less than 1 due to the presence of trace amounts of brassicasteryl and cholesteryl glucosides.

The SG composition of lipids extracted from roots was measured by direct infusion electrospray ionization tandem mass spectrometry (ESI-MS/MS). The resulting profile for wild type roots was ~76% sitosteryl, ~13% campesteryl, ~10% stigmasteryl, ~0.4% brassicasteryl and ~0.1% cholesteryl glucosides, a profile similar to that observed in seeds^16, 25^. *fk-J3158* mutants displayed a reduction in total SGs (58% of wild type levels), with significant decreases in both sitosteryl and campesteryl glucosides (~54% and ~44% of the wild type levels, respectively) (Fig. 1A; Supplementary Table S1). There was also a trend indicating a reduction in stigmasteryl glucosides in this mutant.

The sterol glucosyltransferase mutants examined in this study also exhibit SG profiles that deviate from the wild type. The SG profile of *ugt80A2* mutants showed an overall reduction in total SG, possessing only ~42% of wild-type levels, with sitosteryl, campesteryl, and stigmasteryl glucosides all reduced in roughly equal proportion (~41, 39 and 46% of wild-type levels, respectively) (Fig. 1A,B; Supplementary Table S1). In contrast, total SG was not significantly reduced in *ugt80B1* mutant roots (Fig. 1A). However, the level of stigmasteryl glucosides was only ~56% of what would be expected in wild-type root tissue (Fig. 1A). Therefore, although the overall level of SGs in this mutant are close to wild type, the disproportionate reduction of one particular sterol glucoside results in an altered ratio of sitosteryl:stigmasteryl:campesteryl glucosides in the root tissue of *ugt80B1* mutants (Fig. 1B). Consistent with past findings^15, 16, 25^, the *ugt80A2,B1* double mutant resulted in the most dramatic phenotype with a highly significant reduction in total SG – only ~15% of wild-type levels (Fig. 1A; Supplementary Table S1).

Acyl sterol glucosides (ASGs), which are synthesized from SGs were also measured in each of the mutants and compared to wild type. The *ugt80A2,B1* double mutant displayed a significant reduction in ASG levels at around 66% of WT levels (Supplementary Table S1). However, the single mutants displayed ASG profiles comparable to WT. Levels in *fk-J3158* mutants were even marginally elevated (Supplementary Table S1).

### Epidermal cell patterning in the root

**Figure 2:**
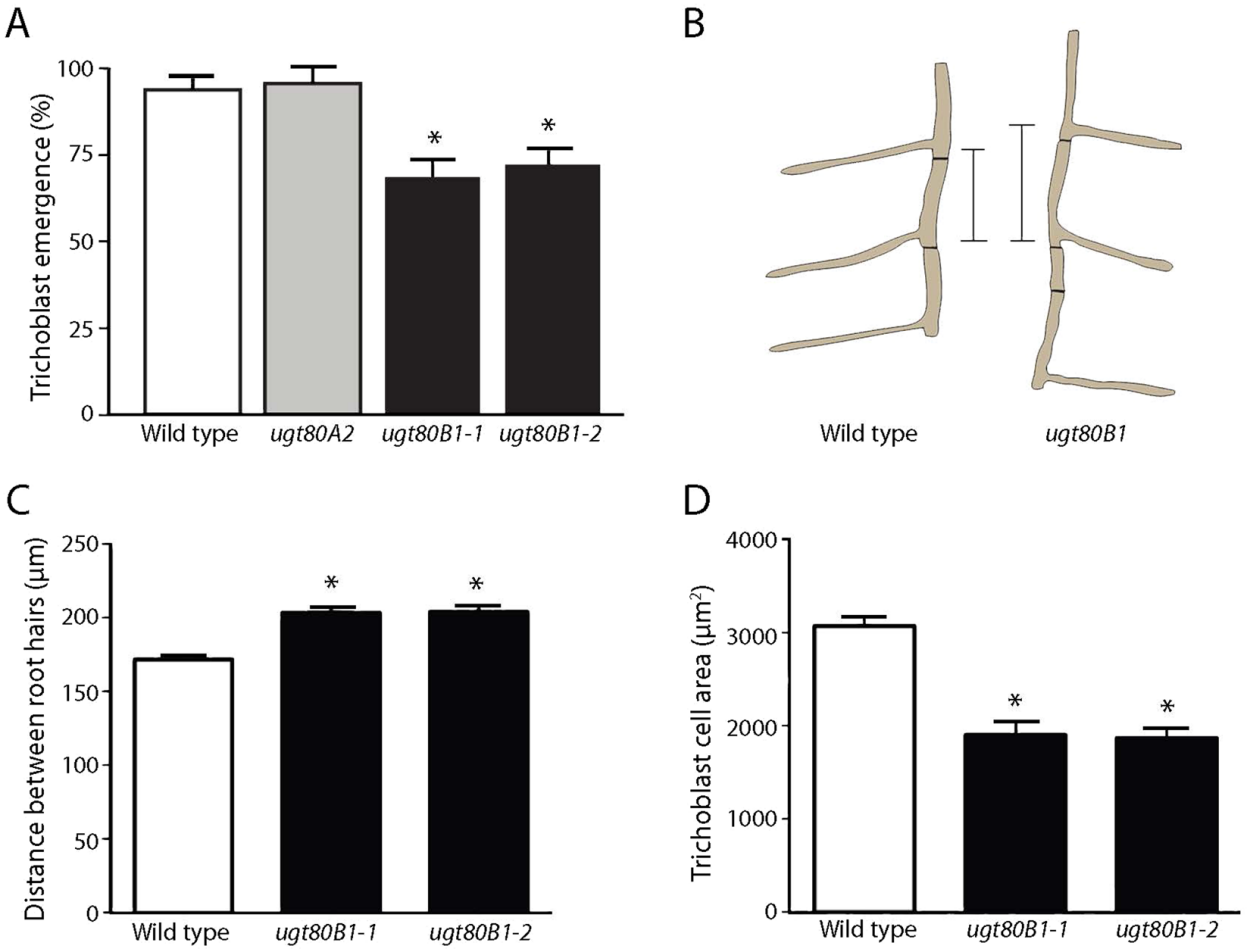
Aberrant epidermal root hair distribution in *ugt80B1* mutants. (**A**) Mean emergence of trichoblasts (%) in H position cell files in the roots of wild type, *ugt80A2 ugt80B1–1*, and *ugt80B1–2* mutant lines. Both *ugt80B1* mutant lines differ significantly from wild type (*p* < 0.001, Student’s T-test). (**B**) Cartoon to illustrate the difference in distance between adjacent root hairs in an H-cell file in wild type and *ugt80B1* mutants (scale bar = 100 μm). (**C**) Mean distance between root hairs in wild type, *ugt80B1–1* and *ugt80B1–2* mutants (P < 0.001, Student’s T-test, n = 32). (**D**) Mean trichoblast cell area in wild type, *ugt80B1–1* and *ugt80B1–2* mutants (P < 0.05, Student’s T-test, n = 19). Asterisks indicate statistical significance, error bars indicate standard error.

Previous work has demonstrated that *ugt80B1* mutants exhibit morphological defects relating to the seed and embryo^15, 16^. In this study we show that *ugt80B1* mutants display aberrant root hair patterning with averages of 31 and 28% of H cells lacking root hairs in *ugt80B1–1* and *ugt80B1–2* respectively (Fig. 2A, Supplementary Table S2). Associated phenotypes include a greater distance between root hairs and lower trichoblast cell area in *ugt80B1* mutants compared to wild type (Fig. 2B–D), indicating that the reduced hair frequency cannot be explained by excessive trichoblast cell elongation.

**Figure 3:**
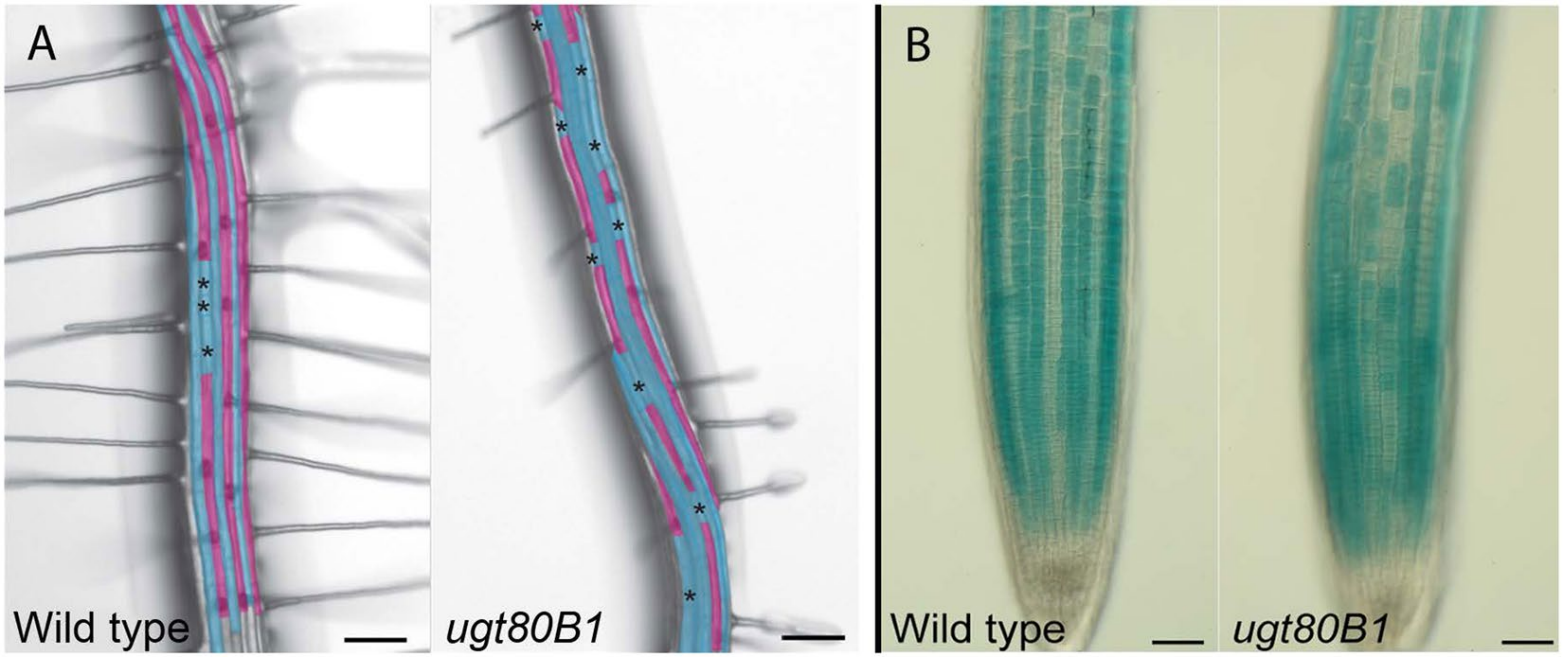
Root hair patterning and GL2 expression are aberrant in ugt80B1 mutants. (**A**) Brightfield microscopy images with false coloration to indicate H (cyan) and N (magenta) cells (scale bar = 100 μm). Black asterisks mark ectopic N cells in an otherwise H cell file. (**B**) The *proGL2:GUS* reporter was used to identify cells expressing GL2 (scale bar = 50 μm). In wild-type roots, cell files in the N position consistently express GL2 whereas those in the H position do not. In *ugt80B1* mutant roots, *proGL2:GUS* expression appears irregular.

This phenotype was visualized by bright-field microscopy images with false coloration to indicate aberrant atrichoblasts in H cell files (Fig. 3A). The presence of these abnormal N cells was further corroborated by the *GL2* transgenic reporter line, *ProGL2:GUS* (Fig. 3B), a reliable indicator of cells committed to become atrichoblasts^27^. In contrast, *ugt80A2* mutants produced no fewer root hairs than wild type plants (Fig. 2A, Supplementary Table S2) and *ugt80A2,B1* double mutants failed to show more severe defects in root patterning than *ugt80B1* single mutants (Supplementary Figure S1).

### *Differential expression of root epidermal cell fate development genes in* ugt80B1

Given the defect in epidermal cell patterning in *ugt80B1* mutant roots, we explored global gene expression patterns in wild-type versus *ugt80B1* 10-day-old seedlings grown hydroponically in the light to enrich for root tissue. Microarray data indicated a total of 565 differentially expressed genes, 480 of which were up-regulated while the remaining 85 were down-regulated (Supplementary Figure S2).

To evaluate these data, the interactive genomic software DAVID was combined with TAIR9 Gene Ontology (GO) description^28^. Dataset interpretation revealed a transcriptional signature described as ‘epidermal cell development’ associated with *ugt80B1* mutants. Several previously characterized regulators of epidermal cell layers were identified including *WER*, and *SCM* (Table S3).

### *Expression of upstream cell fate regulators, SCM and WER, is altered in* ugt80B1 *mutants*

**Figure 4:**
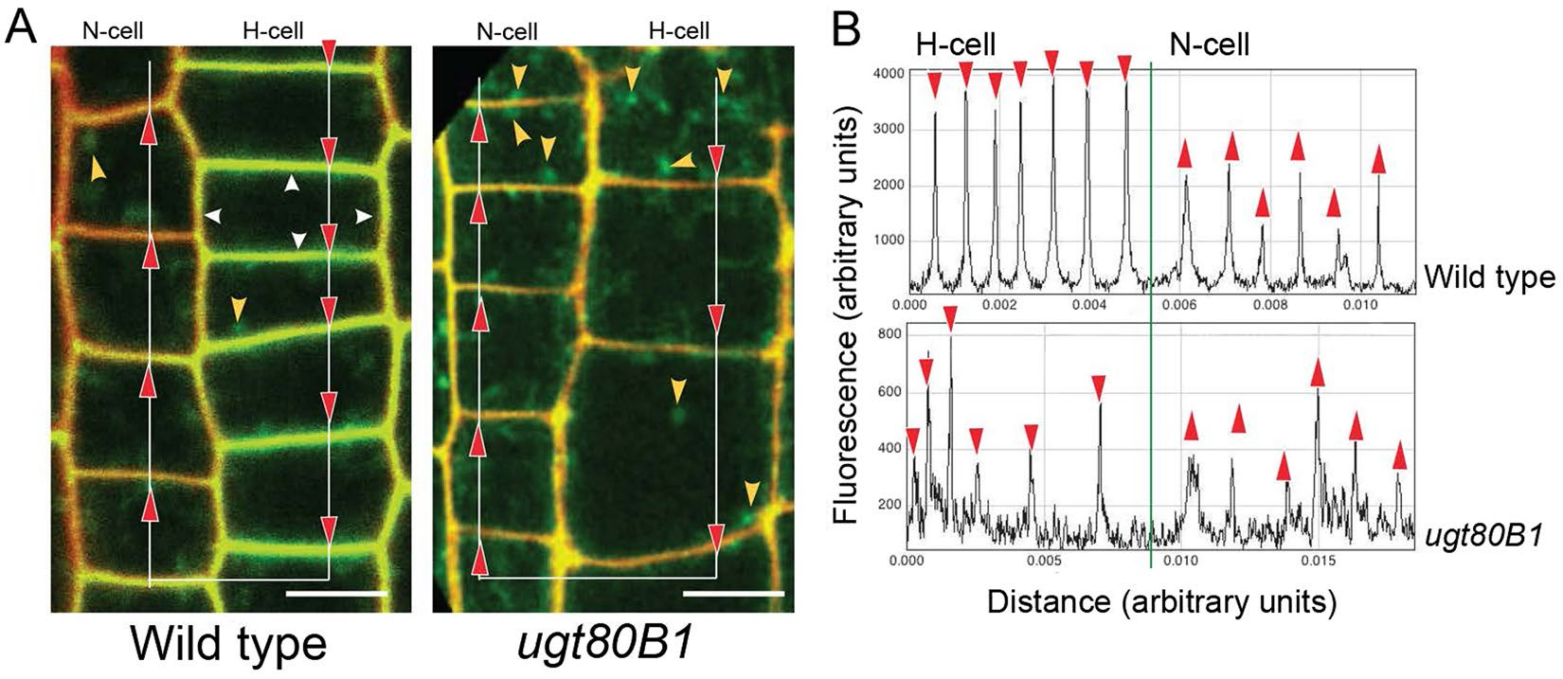
Aberrant subcellular localization of SCM:GFP in *ugt80B1* epidermal cells from the elongation zone of the root. (**A**) Wild-type and ugt80B1 roots expressing proSCM:SC*M:GFP* were stained with propidium iodide (red) to distinguish cell walls from plasma membrane labeled with SCM:GFP (green). Two adjacent cell files from elongation zones are shown. Wild type exhibits clear cell periphery localization of SCM:GFP (white carats) in one of the two cell files, differentiating developing H cells from N cells. In *ugt80B1*, a decrease in cell peripheral localization of SCM:GFP can be seen, and signal is detected in both H and N cell files indicating a loss in cell-type specific accumulation of SCM. An increase in cytoplasmic punctae labeled with SCM:GFP is visible in both cell files in *ugt80B1* (yellow carats). (scale bar = 10 μm) (**B**) Fluorescence intensity transect plots derived from SCM:GFP at the apical-basal plasma membrane juncture of H and N cells were measured in arbitrary fluorescence units for both wild-type and *ugt80B1* (red carats in **A** and **B**).

SCM, a membrane-associated LRR-RLK central to the mediation of positional cues in root epidermal cells^29, 30^, was investigated using confocal microscopy of *SCM:GFP* in the *ugt80B1* and wild-type root epidermis. In the elongation zone of wild-type roots, *SCM:GFP* can be visualized at the cell periphery and preferentially accumulates in H cells (Fig. 4A, first panel), consistent with the current model for root epidermal cell specification. In the mutant background, the reporter indicates that not only is SCM expressed in both H and N cells, but it is no longer restricted to the cell periphery. The second panel of Fig. 4A illustrates the distribution of *SCM:GFP* structures of various sizes in the cytoplasm of the root cells. These results were confirmed by time-lapse confocal imaging with Movie S1 showing that *SCM:GFP* localizes in one cell file in wild type seedlings, clearly demarcating H versus N cells. Movie S2, in contrast, shows a marked decrease in *SCM:GFP* localization at the periphery of the epidermal cells, along with an increase in *SCM:GFP*-labeled structures in the cytoplasm of both cell files in *ugt80B1* mutants.

These patterns of fluorescence signals were quantified using intensity plots along an apical-basal transect of a single cell file spanning the plasma membrane juncture of both H and N cells (Fig. 4B). The plots show that in wild-type seedlings, H cell junctions exhibit fluorescence that is 2- to 4-fold greater than N cell junctions whereas in *ugt80B1* mutants, the intensity of the fluorescence is similar at both H and N cell junctions. The fluorescence intensity plots of the *ugt80B1* cells also displayed numerous low fluorescence peaks presumably corresponding to the SCM structures visible in the cytoplasm in this mutant line.

**Figure 5:**
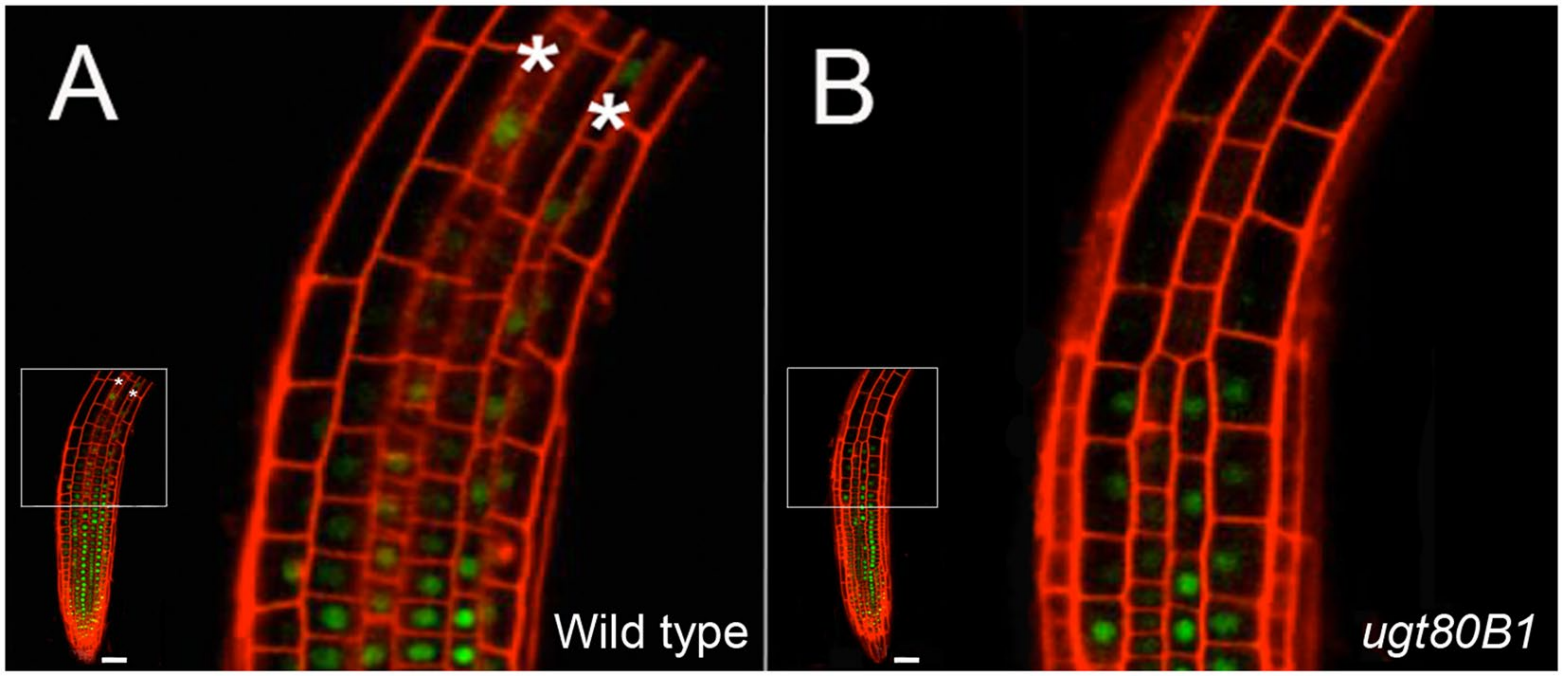
Abnormal expression pattern of *proWER:WER:GFP* in *ugt80B1* mutants. Confocal microscopy imaging of WER:GFP in longitudinal sections of the epidermis from wild-type and *ugt80B1* roots, counter-stained with propidium iodide to visualize cell boundaries (red) (scale bar = 50 μm). White asterisks (**A**) indicate the accumulation of WER:GFP (green) in older/differentiating N cells in wild type. This fluorescence is absent from the corresponding cells in *ugt80B1* mutants (**B**).

WER, a member of the R2R3-MYB transcriptional regulator family and constituent of WER-GL3/EGL3-TTG1, the N cell fate activator complex^23^, acts downstream of SCM. We therefore examined its expression using the *WER* promoter GFP reporter line *proWER:WER:GFP* in the root epidermis. In wild-type roots, this reporter revealed persistent accumulation of WER:GFP in differentiating N cells in the elongation zone (Fig. 5A, white asterisks) whereas in the mutant line, WER:GFP is notably absent from epidermal cells in the elongation zone (Fig. 5B).

### ***UGT80B1*** protein localization

The subcellular localization of the UGT80B1 protein was investigated utilizing C-terminal translational fusions of UGT80B1 to GFP. The *ugt80B1* mutant phenotype was complemented with *pro35S:UGT80B1:GFP* or with *proUGT80B1:UGT80B1:GFP* as observed by rescue of the transparent testa phenotype. The native promoter and 35 S driven fusion proteins were detected as single 95 kDa bands by Western blot using an anti-GFP antibody. Live cell imaging by confocal microscopy revealed that fluorescence arising from plants expressing *proUGT80B1:UGT80B1:GFP* were found to be similar in subcellular localization but displayed lower emission than plants expressing *pro35S:UGT80B1:GFP*. We therefore proceeded to use the *pro35S:UGT80B1:GFP* lines for further live imaging studies.

As suggested from previous reports^31–33^, UDP-Glc:sterol glucosyltransferase was hypothesized to be localized in the ER (endomembrane) and possibly also in the plasma membrane (PM). To test this hypothesis, we visualized UGT80B1:GFP in the subcortical focal plane and could observe features that appeared reminiscent of the highly dynamic cisternae of the ER (Movie S3). We identified the fluorescent bodipy dye ER Tracker (587 excitation nm, 615 nm emission) as a vehicle to test whether fluorescence from UGT80B1:GFP pixels colocalized with pixels from the ER dye and results supported colocalization of the UGT80B1:GFP (Supplementary Figure S3) and the positive control ER reporter Q4:GFP line^34^. Using the same experimental rationale, we employed the membrane reporter FM4–64 (515 nm excitation, 640 nm emission maxima) to visualize colocalization at the PM after short term (5 min) exposure using PLASMAMEMBRANE INTRINSIC PROTEIN2 (PIP2):GFP as positive control, which also revealed satisfactory colocalization (Supplementary Figure S4) to support previous biochemical reports^31–33^.

A plasmolysis experiment depicted in Supplementary Figure S5 lends support to the idea that UGT80B1:GFP was localized in the hechtian strands, connecting the retracting protoplast from the cell wall. However, due to the amount of cytoplasmic ER label, it is difficult to establish whether the PM or ER is the source of the fluorescence. Indeed, the localization of UGT80B1:GFP in the Hechtian strands (Supplementary Figure S5, white carats; Movie S4), may be due to the presence of UGT80B1 in ER and plausibly desmotubule.

## Discussion

The biological function of SGs remains uncertain but the phenotypes exhibited by Arabidopsis lines carrying mutations in the *UGT80B1* (allelic to *transparent testa15*) gene encoding UDP-Glc:sterol-glucosyltransferase are striking^15, 16^. Here, we present further evidence of the important role played by SGs demonstrating that *ugt80B1* mutants display aberrant root epidermal cell patterning. We show that this phenotype is associated with the mislocalization of the upstream cell fate regulator, SCM, which may be indicative of vesicular trafficking defects or interference with targeting to the plasma membrane.

Lipid profile analysis of whole roots showed that *ugt80A2* single mutants and *ugt80A2,B1* double mutants display a significant decrease in SG levels (total and individual) when compared to wild type with the latter presenting the greatest deficiency, evidently due to partial redundancy of the two genes (Fig. 1). However, *ugt80B1* single mutants showed no significant difference in overall SG levels compared to wild type. When individual SGs were examined, the only statistically significant deficiency observed was in stigmasterol glucosides. These results are largely in agreement with the measurements reported by Stucky *et al*.^16^, which showed that UGT80A2 is the dominant enzyme for the production of SGs in seeds while UGT80B1 appears to perform a more specialized role. However, while Stucky *et al*.^16^ describe a structural basis underlying the preference of UGT80B1 for brassicasteryl, cholesteryl and campesteryl glucosides, the data presented here suggest that UGT80B1 preferentially generates stigmasteryl glucosides. This discrepancy suggests that the preferred substrate of UGT80B1 varies over different developmental stages and/or tissue types and confers functional influence on developmental and cellular processes.

**Figure 6:**
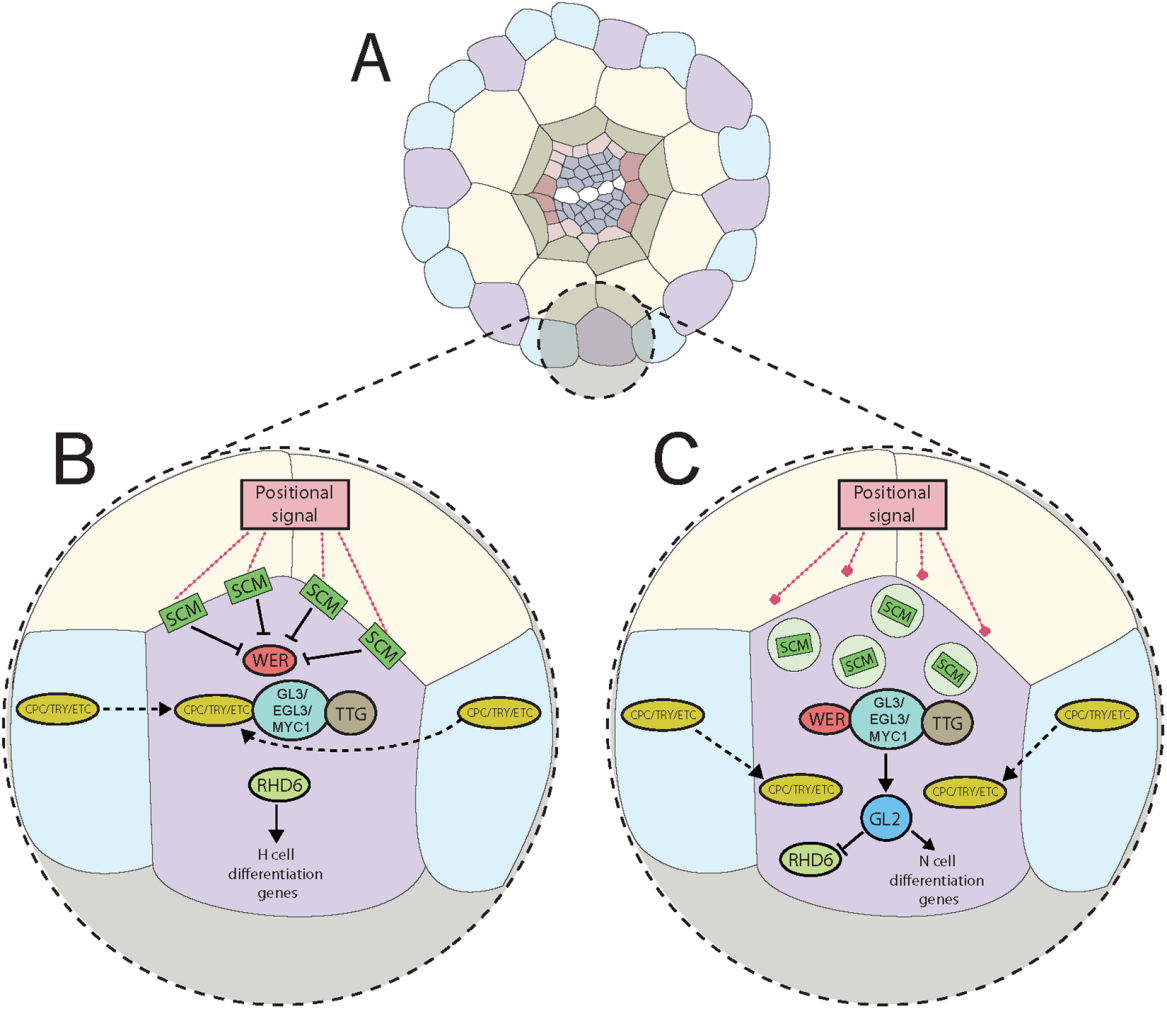
A model to illustrate the downstream effects of SCM mislocalization. (**A**) Diagram depicting cells from a transverse section of an Arabidopsis root. The epidermal layer comprises H cells (purple) and N cells (blue), with H cells positioned over the anticlinal walls of two underlying cortical cells (beige). (**B**) When SCM is localized to the plasma membrane, it mediates the positional signal received from the surrounding cells, consequently inhibiting the expression of WER, allowing CPC/TRY/ETC to form a transcriptional regulatory complex with GL3/EGL3/MYC1 and TTG. RHD6 is free to activate root hair (H) cell differentiation genes. (**C**) If SCM is mislocalized to the cytoplasm, the positional signal is not received. Therefore, WER is left uninhibited and competitively binds to GL3/EGL3/MYC1 and TTG, displacing CPC. This alternative transcriptional regulatory complex activates GL2, which inhibits RHD6 and activates non-hair (N) cell differentiation genes. It appears that *ugt80B1* mutants represent an intermediate stage where SCM is not strictly localized at the cell periphery leading to a reduction in but not an elimination of root hair cells.

As with previous studies examining this pair of mutants^15, 16^, only *ugt80B1* mutants exhibited a visible morphological phenotype – abnormal root epidermal cell patterning – and it was no more severe in the double mutant. In *ugt80B1* mutants, significantly fewer cells in H cell files become trichoblasts. Positional information is crucial for correct root epidermal cell patterning and in wild-type roots, SCM is localized in the plasma membrane^35^, a prime location for receiving signals from underlying cortical cells. However, SCM:GFP visualized in *ugt80B1* mutants is not strictly localized in the cell periphery and there is an increase in cytoplasmic punctae labeled with SCM:GFP. This mislocalization could reduce the reception and transmission of the positional signal by SCM, leading to the disruption of cell fate regulation (Fig. 6). The failure of SCM to target to the plasma membrane could be due to defective trafficking within the endomembrane or a reduction in its PM association. Dalal *et al*.^36^ reported the discovery of a novel stigmasterol binding protein, ROSY1, and postulated that the attachment of this protein to a vesicle depends on the sterol composition of a vesicle’s membrane. Likewise, alterations in the SG composition of vesicle or PM in *ugt80B1* mutants may result in the mislocalization of SCM.

Correct root epidermal cell patterning in Arabidopsis is reliant on interlocking intercellular feedback loops and hormonal regulatory processes^18^. If the expression pattern of any individual cell fate regulator is altered, effects on downstream regulators and, consequently, cell fate determination are inevitable. Indeed, altered expression patterns of both WER:GFP and *ProGL2:GUS* are observed in *ugt80B1* mutants. GL2 is a reliable indicator of atrichoblast cell identity and the aberrant expression of this cell fate regulator in H cell files from *ugt80B1* mutants is likely responsible for the reduction in the formation of root hairs.

Investigations into the localization of the *UGT80B1* protein provide evidence that it has a presence in the PM (Figure S4). These data offer further spatial support for our hypothesis that a defective PM in *ugt80B1* mutants leads to the mislocalization of SCM and ultimately, the reduction in root hair formation. The presence of the *UGT80B1* enzyme in the plasma membrane would underscore the need for a high turnover of sterols to SGs in this membrane compartment and constitutes an interesting avenue for future work in this field.

In summary, we show that abnormal root hair cell patterning in *ugt80B1* mutants is likely the direct result of expression of GL2 in H cell files in this mutant. We propose that the aberrant expression of GL2 is caused by the mislocalization of SCM away from the cell periphery, reducing the capacity of the receptor to mediate positional information to the cell (Fig. 6). Though our lipid profile measurements are insufficient to provide a link between these phenotypes and SG depletion, we believe that it is possible, given the findings of Stucky *et al*.^16^, that localized reductions in specific SGs that could be responsible for the disruption of cell fate regulators in these *ugt80B1* mutants. As with previous work^15^ we speculate as to whether the mislocalization of SCM to the cytoplasm could point to a role for SGs in vesicular trafficking or plasma membrane protein targeting.

## Methods

### Plant material and growth conditions

*Arabidopsis thaliana* lines used in this study were of the Columbia ecotype (Col-0). T-DNA insertion alleles for *ugt80B1–1* (SALK_021175), *ugt80B1–2* (SALK_103581 C), and *ugt80A2* (SALK_020939) were obtained from the Salk collection (Supplementary Figure S6)^37^. The homozygous *ugt80A2,B1* double mutant was constructed by crossing. The *fk-J3158* allele was previously described^26^. DeBolt *et al*.^15^ documented the *ugt80B1* and *ugt80A2* mutants in the Wassilewskija (Ws-0) background. Homozygosity for the Col-0 T-DNA insertions was confirmed by PCR. The transgenic line expressing *ProUGT80B1:GUS* was previously described^15^. Reporter lines for genetic crosses have been described previously: *ProGL2:β-glucuronidase* (*GUS*), *ProWER:WER-green fluorescent protein (GFP)*, *ProSCM:SCM-GFP* ^23, 27, 35^. Reciprocal crosses were performed with *ugt80B1–1* and transgenic lines carrying reporter constructs. Progeny genotypes were confirmed among the F2 by screening for the transparent testa seed phenotype and fluorescence microscopy/GUS staining for reporters and/or PCR with primers for GFP and GUS. Seeds were sterilized using 30% household bleach solution and vernalized for 3 days in 0.15% agar at 4 °C in dark prior to planting.

### Constructs

To generate the CaMV-35S promoter GFP reporter line, the open reading frame for UGT80B1 was amplified from wild-type Col-0 genomic DNA using a DNeasy Plant Mini Kit (QIAGEN) with 5′-ggggacaagtttgtacaaaaaagcaggcttcATGGCTAGTAATGTATTTGATCATCC-3′ and 5′-ggggaccactttgtacaagaaagctgggtCACGCCACCACATGGAAGACAACACT-3′ forward and reverse primers and cloned directly into the destination vector pMDC83 (Curtis and Grossniklaus, 2003). The UGT80B1:GFP reporter line with its native promoter was constructed by PCR amplification using the following: 5′-TCCCCCTGCAGGCCCACCCTAATGTTTGGTCATTTGATGT-3′ and 5′-CGGGGTACCCACCTTTAAAAACTGAATTCAACTAAACAGC-3′. The PCR product was digested with *Sbf*I and *Kpn*I and ligated into the pMDC43 vector at matching sites to replace the CaMV-35S promoter. The UGT80B1 coding sequence was PCR amplified from Col-0 cDNA using 5′-GCTTGGCGCGCCCATGGCTAGTAATGTATTTGATC-3′ and 5′-GGCCTTAATTAATCACACGCCACCACATGGAAGAC-3′. The PCR product was sequenced and digested with *Asc*I and *Pac*I and cloned at the respective sites fusing the cDNA to a C-terminal GFP in the pMDC43 vector containing *proUGT80B1* resulting in the *ProUGT80B1: UGT80B1:GFP:*construct. Both constructs were sequence verified using promoter and gene flanking primers and three nested primers: 5′-CACTGTCCCGTCATTTTG-3′, 5′-GTGCCATTCTTTGGGGAT-3′ and 5′-CGATGTGCAGCCTTTTCT-3′. *Agrobacterium tumefaciens* was tranfected with destination reporter constructs which were transformed into *ugt80B1* plants by floral dipping^38^. Complementation of *ugt80B1* was monitored by restoring testa color^15^. UGT80B1:GFP seedlings examined for subcellular localization were bright homozygous lines identified from the T2 and analyzed in subsequent generations.

### Lipid Extraction and Electrospray Ionization Tandem (ESI-MS/MS) Mass Spectrometry

Seeds were surface-sterilized and sown onto 1.5% plant tissue culture grade agar containing 0.5X Murashige and Skoog medium^39^, followed by stratification for 3 days at 4 °C and transfer to continuous light at 23 °C. At 4–5 days, seedling roots were harvested by cutting with a razor blade and rapid transfer to hot isopropanol (75 °C). Lipids were extracted using a protocol described previously^25^, a modification from the method by Bligh and Dyer^39^. The amount used for mass spectral analysis was 300 μL for *ugt80A2* and *ugt80B1* mutants, and the entire 1 ml sample for the *ugt80A2,B1* and *fk-J3158* mutants. Samples were dried under nitrogen, and a precise amount of standard mix was added. The samples were dissolved in 1.2 ml solvent comprised of chloroform/methanol/300 mM ammonium acetate in water (300/665/35). The internal standard mixture was comprised of 0.3 nmol di12:0-PC, 0.3 nmol di24:1-PC, 0.3 nmol 13:0-lysoPC, 0.3 nmol 19:0-lysoPC, 0.15 nmol di14:0-PE, 0.15 nmol di23:0-PE, 0.15 nmol 14:0-lysoPE, 0.15 nmol 18:0-lysoPE, 0.15 nmol di14:0-PG, 0.15 nmol-di20:0(phytanoyl)-PG, 0.15 nmol 14:0-lysoPG, 0.15 nmol 18:0-PG, 0.15 nmol di14:0-PA, 0.15 nmol di20:0(phytanoyl)-PA, 0.1 nmol di14:0-PS, 0.1 nmol di20:0(phytanoyl)-PS, 0.12 nmol 16:0–18:0-PI, 0.08 nmol di18:0-PI, 0.75 nmol 16:0–18:0-MGDG, 0.65 nmol di18:0-MGDG, 0.18 nmol 16:0–18:0-DGDG, 0.47 nmol di18:0-DGDG. Unfractionated lipid extracts were introduced by continuous infusion into the ESI source on an API 4000 electrospray ionization (ESI) tandem mass spectrometer (Applied Biosystems, Foster City, CA). Samples were introduced by an autosampler (LC MiniPAL, CTC Analytics AG, Zwingen, Switzerland), fitted with an injection loop for the acquisition time, and presented to the ESI needle at 30 µl per min. Targeted methods were employed for analysis of the di20:0(phytanoyl)-PG internal standard, SG, and ASG lipid molecules. Internal standard was detected with a scan for neutral loss of the head group moiety, NL 189.04 (C_3_H_9_O_6_P + NH_3_) in the positive mode. Routine polar lipids were detected as previously described (Xiao *et al*., 2010). For SG detection, a scan for neutral loss of the hexose moiety (NL 197.09, C_6_H_12_O_6_ + NH_3_) was used. ASG lipids were detected with neutral loss scans for the hexose moiety acylated with specified fatty acids (16:0, 18:3, 18:2, 18:1, and 18:0) + NH_3_. Sample runs, mass spectra detection, and data analysis were performed as described by Schrick *et al*.^25^.

### Quantification of epidermal patterning

All measurements were performed at the ‘root hair zone’ as defined previously^40^. This area was approximately one vertical mm from the root tip and all measurements in this area were examined in 5-day-old light grown seedlings. Area measurements for each trichoblast cell used area measurement output after tracing the polygon via the freehand selection tool (ImageJ) and pixel number^2^ converted to μm^2^. Measurements were made using ImageJ and statistical analysis was done using Prism4 (GraphPad, Mountain View, CA) to obtain frequency distributions and significance using Student’s T-test. The distance measurements between trichoblast cells were calculated for wild type and mutant using ImageJ for 6 seedlings each over 1.6 mm length of the root. The measurement was taken between centers of the base of one root hair to the next in the same trichoblast cell file. Percentage emergence of hair and non-hair cells in H and N cell file was calculated after visually analyzing cell files (Supplementary Table S2). Statistical analysis and significance was tested using Mann Whitney U test in the previously mentioned software Prism4.

### Laser scanning confocal microscopy and fluorescence stereomicroscopy

Seeds were germinated on plates containing sterilized 0.5X Murashige and Skoog (MS)^41^ agar for 7–14 d in light conditions at 21 °C in a Conviron Adaptis1000 environmental chamber fitted with a Conviron Arabidopsis light kit (Conviron). For stereomicroscopy, individual seedlings were visualized either in MS agar without removing them from plates and employed both brightfield and fluorescence modes. For confocal microscopy of seedlings, a custom built U-shaped parafilm sandwich was made from 2 layers of parafilm cut with a razor blade to a microscope slide (75 by 25 mm) width and immobilized with vacuum grease to a microscope slide. The U-shaped regions were filled with 0.5x MS media containing 1% agar and sterilized seeds were planted at the open end of the U prior to covering the sandwich with a cover slip and allowing the agar to solidify at a 10° angle. Once mounted, seeds were germinated by placing the mounted slides in a sterilized plate containing a moistened Kimwipe to limit water loss from the slide for 4–7 days in continuous light. Imaging of seedlings in the dark was performed on an Olympus MVX-10 Macro/Stereo fluorescence microscope with a Prior light source and GFP filter (Olympus) and 1X objective employing internal 2X zoom, or an Olympus FV1000 laser scanning confocal microscope using a 20x, 40x or 60x N.A. water-immersion objective equipped with lasers for excitation wavelengths ranging from 405–633 nm. EGFP and ERFP were excited using the 488 nm and 543 nm, respectively and emission spectra were at 509 and 584 nm respectively. Initial image processing was performed using Olympus Fluoview software (Olympus). All further image analysis was performed using ImageJ (W. Rasband, National Institute of Health, Bethesda, MD) software. Colocalization studies of *UGT80B1:GFP* with the ER and membrane were achieved using the fluorescent bodipy dye ER-Tracker (Thermo Fisher Scientific) and the fluorescent reporter dye FM4–64 (Thermo Fisher Scientific)^42, 43^.

## Electronic supplementary material

Supplementary online information

Movie S1

Movie S2

Movie S3

Movie S4
