# Supplementary material for: Positioning of the SCRAMBLED receptor requires UDP-Glc:sterol glucosyltransferase 80B1 in Arabidopsis roots
Source: Sci Rep. 2017 Jul 18;7:5714. doi: 10.1038/s41598-017-05925-6 (PMC5515990; doi:10.1038/s41598-017-05925-6)
Supplement: Supplementary file 1 — Supplementary online information [file 41598_2017_5925_MOESM1_ESM.pdf]

Positioning of the SCRAMBLED receptor requires UDP-Glc:sterol glucosyltransferase 80B1 in *Arabidopsis* roots

Victoria G. Pook[a§], Meera Nair[a§], Kook Hui Ryu[b], James C. Arpin[c], John Schiefelbein[b], Kathrin Schrick[c] and Seth DeBolt[a,†]

Supplementary Information

Supplementary Methods

Supplementary References

Supplementary Tables S1-S3

Supplementary Figures S1-S6

Supplementary Movies S1-S4

Supplementary materials and methods

Quantification of epidermal patterning All measurements were performed at the 'root hair zone' as defined earlier[1]. This area was approximately one vertical mm from the root tip and all measurements in this area were examined in 5-day old light grown seedlings. Area measurement for each trichoblast cell used area measurement output after tracing the polygon via the freehand selection tool (ImageJ) and pixel number^2 converted to μm^2. Measurements were made using ImageJ and statistical analysis was done using Prism4 (GraphPad) to obtain frequency distributions and significance using Students T-test. The distance measurements between trichoblast cells were calculated for wild-type and mutant using ImageJ for 6 seedlings each over 1.6 mm length of the root. The measurement was taken between centers of the base of one root hair to the next in the same trichoblast cell file. Percentage emergence of hair and non-hair cells in H and N cell file was calculated after manually analyzing the cell files for a minimum of 40 seedlings. Statistical analysis and significance was tested using Mann Whitney U test on Prism4 (GraphPad).

Global analysis of genes responding to ugt80B1 *ugt80B1* and wild-type seeds were germinated and grown in liquid culture with 100 mg of seed in exactly 100 mL of half strength MS media in each 250 mL conical flask in triplicate to eliminate secondary effects due to development and environment. These plants were grown under long-day conditions (16-h/8-h photoperiod) for 10 days and all experiments were harvested within 5 minutes to avoid secondary effects of circadian clock related transcripts. The approach used also ensured adequate seedling material (100 mg = approximately 5500-6000 seedlings) for each RNA extraction, added experimental robustness by the large number of individuals in each sample and minimized any minor developmental transition effects. Messenger RNA was extracted using QIAGEN RNeasy mini kit for RNA extraction as per manufacturer's instructions (QIAGEN). Since *UGT80B1* is expressed in all plant tissues (see eFP-analysis of At1g43620[2]), 10-day post germination (dpg) seedlings were used for transcriptional profiling. Hybridization and gene chip analysis was performed as a fee for service at the University of Kentucky Microarray Core Facility. The data was then LOESS normalized. A P-Value was established at <0.01. A two–fold shift in gene expression was then used to qualify genes differentially expressed. We also applied DAVID analysis to the dataset, which essentially provides an enrichment framework to systematically map genes in the given list to the associated biological annotation and highlight the most enriched or overrepresented biological annotation. The methods used for the DAVID analysis are defined in Huang et al.[3].

Laser scanning confocal microscopy and fluorescence stereomicroscopy Seeds were germinated on plates containing sterilized 0.5 X MS agar for 7–14 d in light conditions at 21°C in a Conviron Adaptis1000 environmental chamber affixed with a Conviron Arabidopsis light kit (Conviron). In the case of stereomicroscopy, individual seedling were visualized either in MS agar without removing them from plates and employed both brightfield and fluorescence modes. For confocal microscopy, a custom built U-shaped parafilm sandwich was created for growing seedlings. Here, 2-layers of parafilm were cut with a razor blade to a microscope slide width U-shape and then sandwiched with vacuum grease on a microscope slide. The U-shaped regions was filled with 0.5 x MS Agar media slide and sterilized seeds were planted at the open end of the U prior to covering the sandwich with a cover slip and allowing the agar to set on a 10° gradient. Once mounted, specimens were grown in a sterilized plate containing a moistened kimwipe to limit water loss from the slide for 4-7 days in 24 hr light. Seedlings were then carefully placed on the confocal microscope stage and imaged in darkness. Imaging was performed on an Olympus MVX-10 Macro/Stereo fluorescence microscope with a Prior light source and GFP filter (Olympus) and 1X objective employing internal 2X zoom, or an Olympus FV1000 laser scanning confocal microscope using a 20x, 40x or 60x N.A. water-immersion objective. The microscope is equipped with lasers

for excitation wavelengths ranging from 405–633 nm and EGFP and ERFP were excited using the EGFP (488 nm) and ERFP (543 nm) setting in the Olympus Fluoview software (Olympus). Initial image processing was performed using Olympus Fluoview software (Olympus). All further image analysis was performed using ImageJ (W. Rasband, National Institute of Health, Bethedsa, MD) software.

Plasmolysis 5-day-old transgenic seedlings expressing *35S::UGT80B1-GFP* were treated with 0.8M mannitol for 2 hours with gentle shaking. The seedlings were stained with 10µg/ml propidium iodide prior to imaging the cotyledons.

Supplementary References

1. Schiefelbein, J. W. & Somerville C. (1990) Genetic control of root hair development in *Arabidopsis thaliana*. *Plant Cell* **2,** 235-243.
2. Winter, D. *et al.* (2007) An "Electronic Fluorescent Pictograph" browser for exploring and analyzing large-scale biological data sets. *PLoS ONE* **2**, e718.
3. Huang, D. W., Sherman, B. T. & Lempicki, R. A. (2008) Systematic and integrative analysis of large gene lists using DAVID bioinformatics resources. *Nat. Protocols* **4**, 44-57.
4. Dinneny, J. R., Long, T. A., Wang, J. Y., Jung, J. W., Mace, D., Pointer, S., Barron, C., Brady, S. M., Schiefelbein, J. & Benfey, P.N. (2008) Cell identity mediates the reesponse of Arabidopsis roots to abiotic stress. *Science* **320**, 942-945.
5. Won, S-K., Lee, Y-J., Lee, H-Y., Heo, Y-K., Cho, M., & Cho, H-T. (2009) cis-Element- and transcriptome-based screening of root hair-specific genes and their functional characterization in Arabidopsis. *Plant Physiol.* **150**, 1459-1473.

Table S1. SG and ASG quantification in roots. Mass spectral signals are shown as percentages of the total signal that included >95% total routine polar lipids for each sample. Averages are indicated for n=4, with standard deviations in parentheses. Significant decreases from Col wild type (WT) are indicated by a single asterisk (P < 0.05) or double asterisk (P < 0.001).

	**WT Col**	***ugt80A2***	***ugt80B1***	***ugt80A2,B1***	***fk-J3158***
**Total SGs**	2.283 (0.170)	0.948** (0.058)	1.900 (0.305)	0.348** (0.086)	1.322** (0.268)
**sitosteryl**	1.745 (0.148)	0.714** (0.034)	1.521 (0.246)	0.263** (0.066)	0.951** (0.193)
**stigmasteryl**	0.296 (0.019)	0.136** (0.014)	0.165** (0.021)	0.039** (0.008)	0.242 (0.058)
**campesteryl**	0.231 (0.010)	0.091** (0.011)	0.204 (0.036)	0.036** (0.011)	0.101** (0.019)
**brassicasteryl**	0.008 (0.000)	0.005** (0.001)	0.007 (0.001)	0.003** (0.001)	0.019 (0.004)
**cholesteryl**	0.002 (0.000)	0.002 (0.001)	0.003 (0.001)	0.007 (0.003)	0.009 (0.003)
**Total ASGs**	1.525 (0.281)	1.421 (0.333)	1.527 (0.538)	1.004* (0.295)	2.384 (0.569)
**Total 16:0**	0.598 (0.085)	0.582 (0.088)	0.614 (0.256)	0.242* (0.036)	0.849 (0.235)
**16:0 sitosteryl**	0.451 (0.068)	0.409 (0.053)	0.480 (0.198)	0.157* (0.024)	0.563 (0.138)
**16:0 stigmasteryl**	0.049 (0.009)	0.050 (0.007)	0.033 (0.013)	0.020* (0.005)	0.132 (0.046)
**16:0 campesteryl**	0.086 (0.012)	0.112 (0.028)	0.092 (0.041)	0.040** (0.007)	0.113 (0.044)
**16:0 brassicasteryl**	0.005 (0.001)	0.005 (0.002)	0.003 (0.002)	0.007 (0.005)	0.023 (0.015)
**16:0 cholesteryl**	0.007 (0.003)	0.006 (0.001)	0.006 (0.003)	0.018 (0.008)	0.018 (0.006)
**Total 18:3**	0.238 (0.057)	0.259 (0.069)	0.236 (0.069)	0.099* (0.035)	0.376 (0.051)
**18:3 sitosteryl**	0.174 (0.040)	0.192 (0.050)	0.180 (0.054)	0.044* (0.013)	0.230 (0.050)
**18:3 stigmasteryl**	0.027 (0.010)	0.028 (0.007)	0.018 (0.004)	0.008* (0.004)	0.057 (0.012
**18:3 campesteryl**	0.029 (0.005)	0.032 (0.010)	0.030 (0.010)	0.030 (0.010)	0.064 (0.015)
**18:3 brassicasteryl**	0.003 (0.001)	0.003 (0.001)	0.003 (0.000)	0.004 (0.004)	0.009 (0.002)
**18:3 cholesteryl**	0.005 (0.003)	0.004 (0.001)	0.005 (0.002)	0.013 (0.006)	0.016 (0.002)
**Total 18:2**	0.343 (0.068)	0.272 (0.079)	0.360 (0.112)	0.242 (0.082)	0.560 (0.136)
**18:2 sitosteryl**	0.257 (0.046)	0.204 (0.056)	0.283 (0.086)	0.142* (0.042)	0.389 (0.110)
**18:2 stigmasteryl**	0.029 (0.006)	0.029 (0.009)	0.024 (0.007)	0.016* (0.004)	0.071 (0.025)
**18:2 campesteryl**	0.044 (0.014)	0.031 (0.010)	0.042 (0.015)	0.046 (0.017)	0.061 (0.011)
**18:2 brassicasteryl**	0.002 (0.001)	0.003 (0.001)	0.004 (0.001)	0.006 (0.002)	0.013 (0.002)
**18:2 cholesteryl**	0.011 (0.009)	0.005 (0.002)	0.008 (0.004)	0.032 (0.020)	0.026 (0.007)
**Total 18:1**	0.259 (0.080)	0.237 (0.087)	0.216 (0.071)	0.199 (0.086)	0.369 (0.105)
**18:1 sitosteryl**	0.183 (0.038)	0.173 (0.062)	0.162 (0.053)	0.084* (0.029)	0.200 (0.064)
**18:1 stigmasteryl**	0.026 (0.021)	0.016 (0.005)	0.011 (0.004)	0.036 (0.022)	0.060 (0.019)
**18:1 campesteryl**	0.032 (0.010)	0.037 (0.015)	0.028 (0.010)	0.024 (0.010)	0.053 (0.015)
**18:1 brassicasteryl**	0.008 (0.011)	0.004 (0.002)	0.004 (0.002)	0.020 (0.011)	0.022 (0.010)
**18:1 cholesteryl**	0.010 (0.005)	0.008 (0.003)	0.010 (0.004)	0.035 (0.018)	0.033 (0.009)
**Total 18:0**	0.086 (0.033)	0.071 (0.028)	0.102 (0.034)	0.223 (0.074)	0.231 (0.009)
**18:0 sitosteryl**	0.037 (0.011)	0.035 (0.013)	0.049 (0.019)	0.072 (0.028)	0.059 (0.019
**18:0 stigmasteryl**	0.007 (0.003)	0.006 (0.002)	0.005 (0.002)	0.011 (0.006)	0.015 (0.008)
**18:0 campesteryl**	0.014 (0.008)	0.011 (0.004)	0.017 (0.010)	0.080 (0.028)	0.086 (0.019)
**18:0 brassicasteryl**	0.003 (0.001	0.003 (0.001)	0.003 (0.001)	0.008 (0.004)	0.011 (0.004)
**18:0 cholesteryl**	0.025 (0.014)	0.017 (0.008)	0.028 (0.008)	0.052 (0.018)	0.060 (0.028)
**Total Routine Polar Lipids**	95.638 (1.206)	97.595 (0.389)	96.354 (0.965)	98.581 (0.347)	96.224 (0.843)

Table S2. Epidermal cell counts from Arabidopsis roots. Number of H- and N cells in H- and N cell files in wild type, *ugt80B1-1, ugt80B1-2* and *ugt80A2* mutants.

Wild type				*ugt80B1-1*				*ugt80B1-2*				*ugt80A2*			
H cell file		N cell file		H cell file		N cell file		H cell file		N cell file		H cell file		N cell file	
H	N	H	N	H	N	H	N	H	N	H	N	H	N	H	N
5	0	0	7	5	4	0	9	5	0	0	10	4	0	0	6
4	1	0	7	4	4	0	10	5	4	0	8	4	0	0	6
3	1	0	5	4	2	0	13	2	4	1	7	4	0	0	7
3	2	0	6	3	0	4	5	4	2	0	5	3	1	0	5
6	0	0	7	5	0	0	6	5	0	0	7	4	0	0	5
4	0	0	7	3	1	0	6	6	1	0	6	5	0	0	5
6	1	0	5	1	4	0	7	4	2	1	7			0	6
8	0	0	4	4	1	0	5	5	0	0	6			0	6
5	0	0	7	4	1	0	6	4	2	0	8				
5	0	0	8	3	2	3	1	4	3	0	7				
6	1	0	10	5	0	0	5	5	2	0	6				
8	0	0	7	5	1	0	6	5	2	0	7				
3	1	0	5	4	2	0	6	5	0	0	7				
5	0	0	8	4	0	2	2	3	2	1	6				
5	0	0	6	4	0	0	6	5	1	0	6				
4	0	0	8	3	2	0	4	3	3	0	6				
2	2	0	7	1	5	0	6	5	1	0	8				
3	0	0	7	4	1	0	6	5	0	0	5				
4	0	0	6	4	2	1	4	3	1	0	5				
4	0	0	7	5	0	0	3	2	4	2	4				
4	0	0	5	2	5	0	6	3	1	0	8				
8	0	0	6	4	0	0	5	5	2	0	7				
4	0	0	5	4	4	0	5	6	2	0	5				
4	0	0	4	6	2	5	2	4	2	0	5				
4	0	0	9	0	6	0	6	4	5	4	3				
4	0	0	6	3	5	0	7	4	1	0	6				
4	0	0	5	4	0	1	5	6	3	0	9				
5	0	0	5	3	0	0	6	5	3	0	6				
4	0	0	6	5	1	0	5	4	1	0	9				
4	1	0	4	3	5	0	6	4	0	1	6				
5	0	0	4	3	1	0	7	4	0	1	5				
3	0	0	6	5	3	1	6	4	2	0	7				
8	0	0	7	3	3	2	5	4	5	0	9				
5	1	0	6	3	4	0	7			0	8				
4	2	0	5	5	1	0	4			0	6				
6	0	0	5	4	3	1	3			0	5				
5	0	0	8	4	1	0	4			0	8				
5	0	0	8	4	0	3	5			0	9				
5	0	0	9	3	1	0	7			2	3				
5	0	0	7	6	0	0	5			1	5				
5	0	0	5	2	7	0	8			0	7				
5	0	0	6			2	5			3	4				
5	0	0	5			0	7			0	7				
5	0	0	8			0	9			0	6				
5	0	0	5			3	3			0	6				
4	1	0	8			0	8			2	11				
6	0	0	7			0	9			0	9				
8	0	0	8			1	2								
7	0	0	10			0	4								
5	2	0	7			0	4								
4	0	0	6			0	8								
4	0	0	7			0	7								
9	0	0	11												
4	1	0	8												
		0	11												

Table S3. Differentially expressed genes related to cell fate and patterning. Microarray analysis of *ugt80B1* versus wild-type seedlings was used to identify mRNA expression differences. See Figure S3.

#	TAIR ID	Gene Name	Δ	Functional Annotation	Reference
1	At2g37260	*WEREWOLF*	+ 2.95	Transcription factor, epidermal cell fate specification, seed coat development	
2	At1g11130	*SCRAMBLED*	+ 2.02	Signal transduction, Regulates expression of GLABRA2, CAPRICE, WEREWOLF, and ENHANCER OF GLABRA3	
3	At1g63650	*ENHANCER OF GLABRA3*	+ 3.10	Epidermal cell fate	Dinneny et al.[4]
4	At3g13840	*SCARECROW*	+ 4.29	Regulation of transcription	Dinneny et al.[4]
5	At5g35190	*EXTENSIN -LIKE PROTEIN*	+ 2.24	Cell wall organization, proline-rich extension-like family protein	
6	At3g10710	*PECTINESTERASE FAMILY PROTEIN*	+ 3.37	Cell wall modification	Won et al.[5]
7	At3g56980	*BHLH039/ORG3*	+ 3.77	Transcription factor	
8	At5g60520	*LATE EMBRYONIC ABUNDANT PROTEIN - I*	+ 2.33	Late embryogenesis abundant protein-related	
9	At4g19690	*Fe(II) TRANSPORT PROTEIN*	+ 6.28	Cellular iron ion homeostasis, response to bacterium and ion transport (Iron, Cadmium, manganese and zinc)	
10	At3g58060	CATION EFFLUX FAMILY PROTEIN	+ 2.31	Cation transport membrane	
11	At4g37070	*PATATIN-LIKE PROTEIN*	+ 2.40	Lipid metabolic process, Acyl transferase/acyl hydrolase/lysophospholipase	Won et al.[5]
12	At4g23690	*PUTATIVE DISEASE RESISTANCE PROTEIN*	+ 2.32	Defense response, ligan biosynthetic process, disease resistance-responsive family protein / dirigent family protein	Won et al.[5]
13	At5g67400	*PEROXIDASE*	+ 4.00	Oxidative stress response	Won et al.[5]
14	At3g01190	*PEROXIDASE 27*	+ 2.12	Oxidative stress response	Won et al.[5]
15	At2g39690	*UNKNOWN PROTEIN*	+ 2.19	Unknown, mitochondrion	Won et al.[5]
16	At1g64780	*AMMONIUM TRANSPORTER*	- 2.51	Transporter	Won et al.[5]

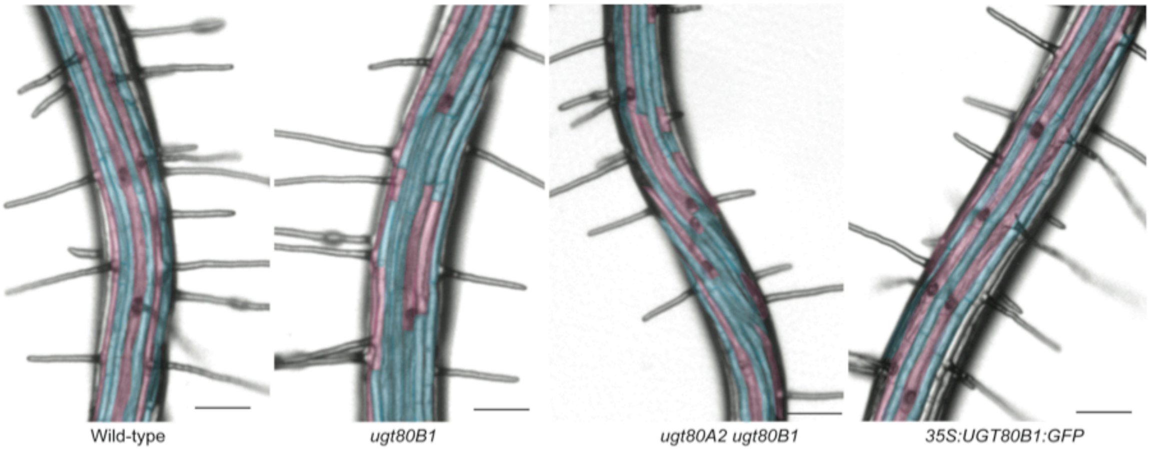

Figure S1. Visual comparison of *ugt80A2, ugt80B1, ugt80A2ugt80B1* and *35S:UGT80B1:GFP* Arabidopsis roots using stereomicroscopy. Root epidermal patterning in *ugt80A2* and *35S:UGT80B1* was indistinguishable in comparison to wild type, whereas *ugt80B1* displays a visible epidermal patterning defect. The *ugt80A2 ugt80B1* double mutant displays epidermal defects that are no more severe than those seen in the *ugt80B1* single mutant. Scale bars = 100 µm.

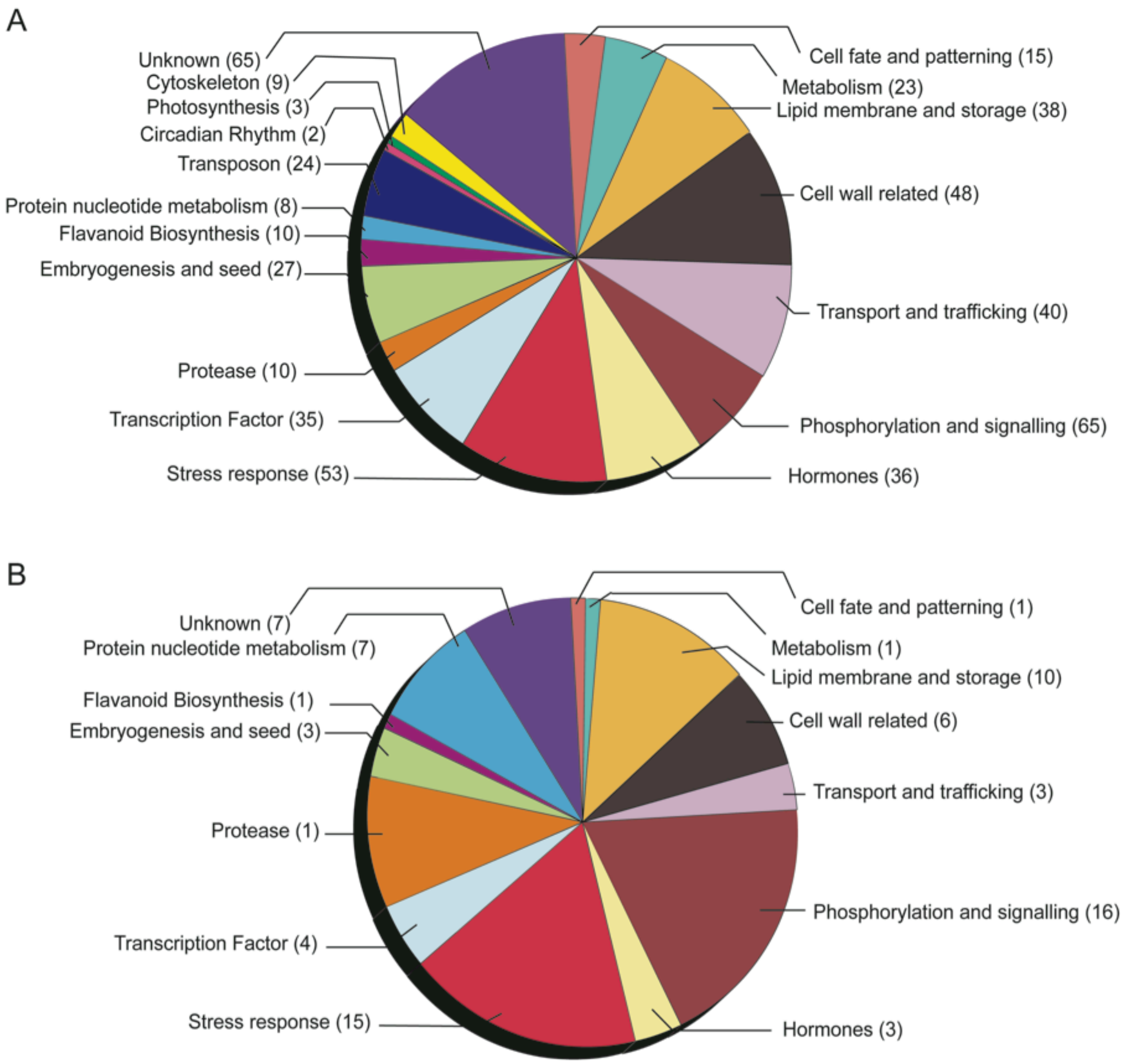

Figure S2. Pie charts illustrating *ugt80B1* differentially expressed genes in comparison to wild type. **A.** Up-regulated genes. **B.** Down-regulated genes. The number of genes with the given annotation are indicated in parentheses. See Table S3 for a list of genes from the "Cell fate and patterning" category.

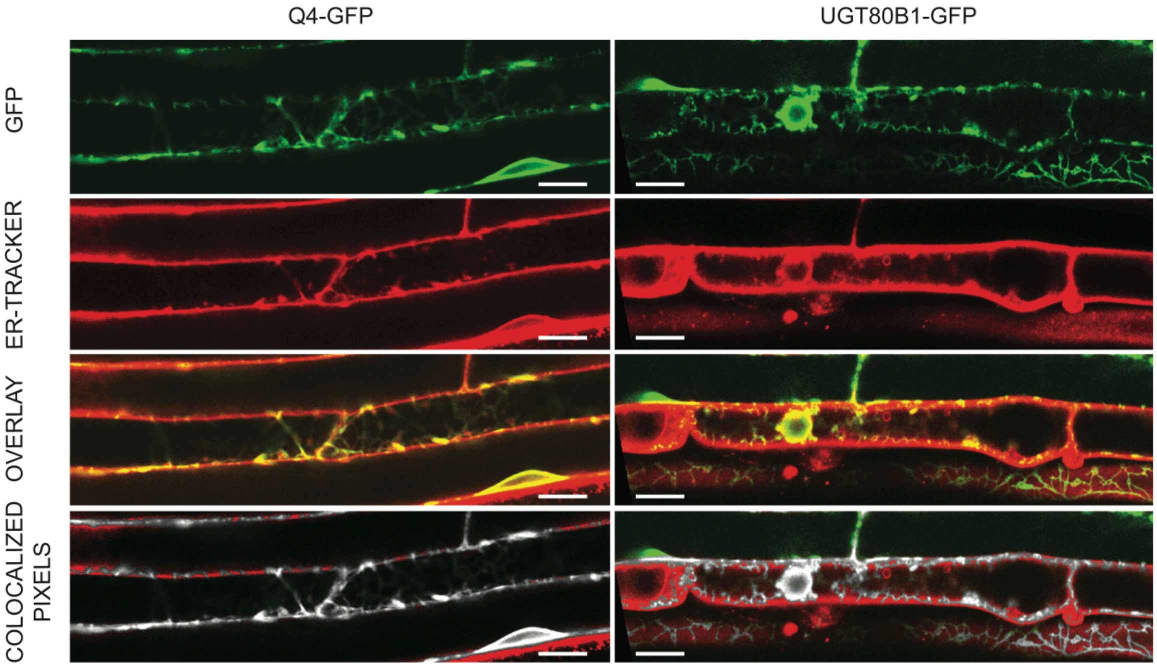

Figure S3. UGT80B1:GFP colocalizes with the fluorescent bodipy dye ER Tracker.
Confocal microscopy of the sub-cortical focal plane in root epidermal cells of 5-day-old seedlings expressing UGT80B1:GFP. From top to bottom: localization of UGT80B1:GFP, localization of the fluorescent bodipy dye ER-Tracker, overlay of UGT80B1:GFP and ER-Tracker, and the bottom panel shows the colocalized pixels in grey. Q4-GFP serves as a positive control that is ER-localized. Scale bar = 10 μm.

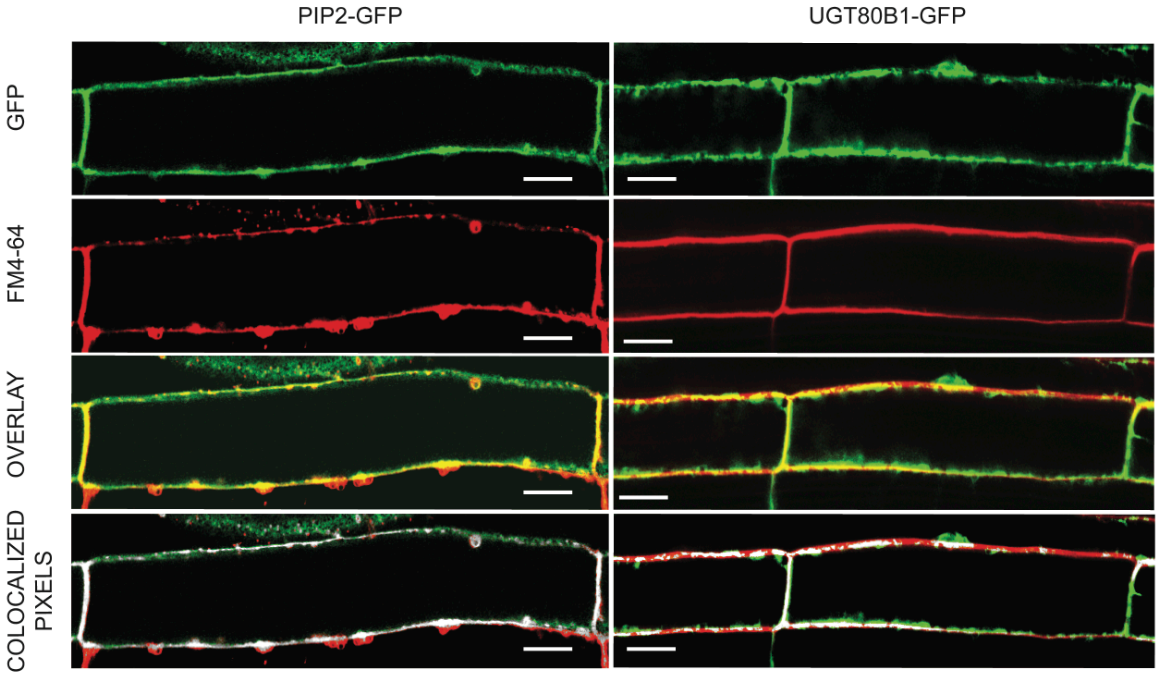

Figure S4. UGT80B1:GFP colocalizes with the membrane reporter FM4-64.
Confocal microscopy of the plasma membrane (PM) in root epidermal cells of 5-day-old seedlings expressing UGT80B1:GFP. From top to bottom: localization of the UGT80B1:GFP signal, localization of the fluorescent reporter dye FM4-64, overlay of UGT80B1:GFP and FM4-64, and the bottom panel shows the colocalized pixels in grey. PIP-GFP serves as a positive control that is PM localized. Scale bar = 10 µm.

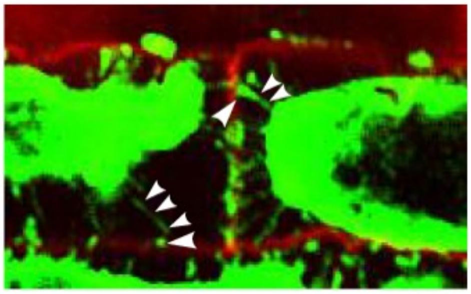

Figure S5. Membrane localization of UGT80B1:GFP reporter in root epidermal cells.
Root epidermal cells expressing UGT80B1:GFP (green), stained with propidium iodide (red) to visualize the cell wall, shown after plasmolysis with 0.8 M mannitol. UGT80B1:GFP labeled punctate dots attached to cell wall with Hechtian strands (white carats).

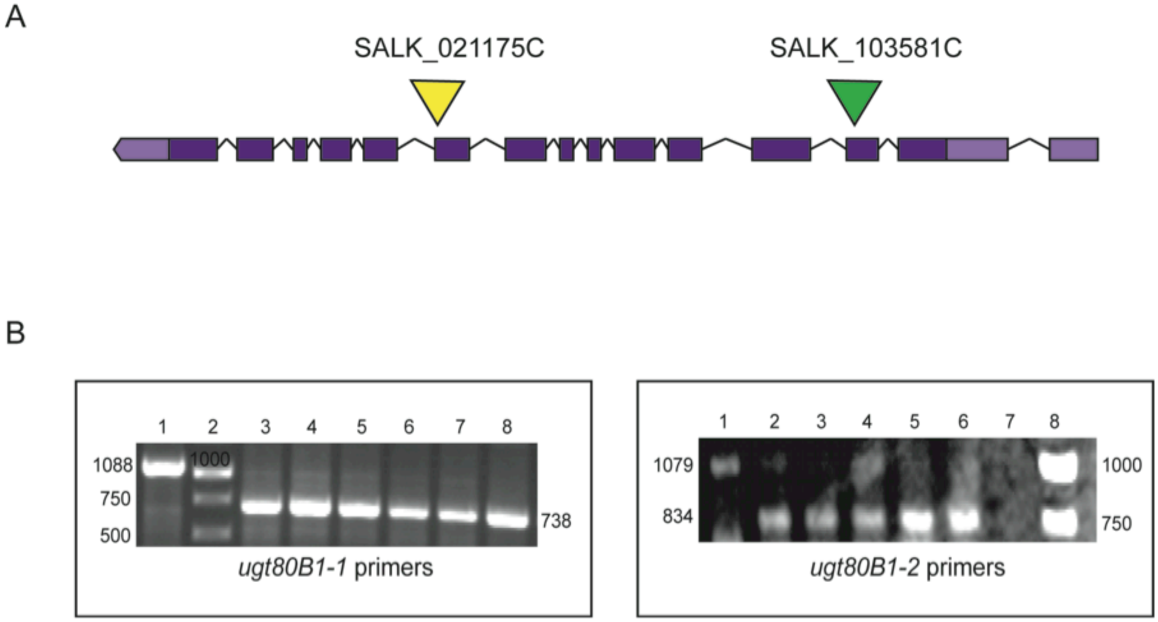

Figure S6. T-DNA insertion alleles for *UGT80B1.* A) Arrowheads indicate positions of T-DNA insertions in *ugt80B1* alleles SALK_021175C and SALK_103581C. **B)** Homozygosity of T-DNA insertions was confirmed by PCR: Left panel lanes: 1 (wild-type band), 2 (molecular-weight size marker), 3-8 (single band in the homozygous mutants at 738 bp); Right panel lanes: 1 (wild-type band), 2-6 (single band in the homozygous mutants at 834 bp), 7 empty lane, and 8 (molecular-weight size marker).

Movie S1. ***SCM:GFP* localization in wild-type root epidermal cells.**
Confocal microscopy time-lapse movie of *SCM:GFP* in wild-type root epidermal cells focusing on two adjacent epidermal cell files. Stable preferential accumulation of *SCM:GFP* on the periphery of cells of one cell file (H-cell) than other (N-cell) is evident. Images were captured at every 10 seconds and stacked using ImageJ. Movie played at 7 frames per second. Scale bar 10 µm.

Movie S2. ***SCM:GFP* localization in *ugt80B1* root epidermal cells.**
Confocal microscopy time-lapse movie of *SCM:GFP* in *ugt80B1* root epidermal cells focusing on two adjacent epidermal cell files. No preferential accumulation was observed and decreased peripheral accumulation of *SCM:GFP* can be seen in cells of both cell files, in addtion to increased punctae in cytoplasm of *ugt80B1* cells. Images were captured at every 10 seconds and stacked using ImageJ. Movie played at 7 frames per second. Scale bar 10 µm.

Movie S3. UGT80B1:GFP localizes to the ER.
Confocal microscopy of the ER in epidermal cells of dark grown hypocotyl regions in *Pro35S:UGT80B1:GFP* expressing plants. Static cortical ER is seen in the background as a meshwork and highly dynamic cisternae and tubular ER strands are visualized in the foreground. Images captured at every 10 second was stacked using ImageJ and movie played at 7 frames per second. Scale bar 10 µm.

Movie S4. **UGT80B1:GFP labeled punctate tether to cell wall via stable Hechtian strands after plasmolysis.**
Confocal microscopy of 5-day-old seedlings expressing *Pro35S:UGT80B1:GFP* after plasmolysis with 0.8 M mannitol. Time-lapse focusing on the tubular UGT80B1:GFP (green) labeled ER structures showing coordinated movement from adjacent cells. Cell wall counter stained with propidium iodide (red) to add contrast. Images captured every 12 seconds, stacked using ImageJ and plays at 7 frames per second. Scale bar 50 µm.
